# The Effects of Loading Conditions on the Behaviour of Fixed-Ended Plain Channel Columns

**DOI:** 10.3390/ma13061441

**Published:** 2020-03-21

**Authors:** Noorfaizal Yidris, Nur Hazwani Isham, Ezanee Gires, Ayad Mutafi

**Affiliations:** Department of Aerospace Engineering, Universiti Putra Malaysia, Selangor 43400, Malaysia; waniisham94@gmail.com (N.H.I.); ezanee@upm.edu.my (E.G.); ayad2motafi@yahoo.co.uk (A.M.)

**Keywords:** thin-walled structures, plain channel, post-buckling, finite element analysis, loading conditions

## Abstract

The compressive behaviour of column members can be considerably affected by local buckling, material yielding and local end conditions. In this paper, the effects of the loading conditions at the ends of plain channel section columns subjected to uniformly compressed loading, and fixed conditions at the column ends with respect to global rotations, was examined. Finite element simulation was employed to look at the post-buckled response of thin-walled, plain channel section columns that covered the complete loading history of the compression columns from the onset of elastic local buckling through the nonlinear elastic and elastoplastic post-buckling phases of behaviour to final collapse and unloading. Two types of loading conditions were considered: the first was one that has been used practically in tests whereby one end is loaded with a moving top platen while the other end is fixed at the lower platen, but, for the second loading condition, both ends were loaded with equally moving top and lower platens. These two conditions were shown to lead to quite different characteristic interactive responses of the columns due to mode jumping in the buckling mode for the locally rotationally constrained case.

## 1. Introduction

There are many aspects which influence the carrying capacity of thin-walled sections; material yielding can be the cause of failure for thin-walled, short length sections. The influence of local buckling and material yielding on the struts was studied by Yidris et al. [1,2] and Loughlan et al. [3,4] for the case of plain channel, I- and box-section struts using the finite element post-local buckling procedure. The longitudinal membrane stresses across the plain channel section strut at the buckle crest change and redistribute as a result of the local buckling of the section walls and, due to geometrical imperfections and material yielding, the strength capacity of the plain channel struts is further reduced. Loughlan et al. [3,4] gave insight into the effects of local buckling on the compression of I- and box-section struts and the growth of material yielding from the point of the first yield through the nonlinear elastoplastic post-buckling phase of behaviour to final collapse at zero compressional stiffness and the subsequent unloading phase of behaviour. In these works, it has been shown that the compressional stiffness of the struts is noticeably reduced at the instant of local buckling. After local buckling, the compressional stiffness continues to reduce gradually as a result of local form change with increased load.

For longer length sections, the intermediate or global buckling effects, such as distortional buckling or the overall modes of torsional, flexural or torsional–flexural buckling, and different geometrical imperfection mode shapes may be of influence. The column interaction of local buckling with the overall column bending of lipped channel, I-sections and plain channel sections was investigated by Loughlan et al. [5], Loughlan and Yidris [6,7,8,9] and Yidris et al. [10]. In these works, the global elastic Euler buckling loads have been shown to reduce as a result of the weakening effects of local buckling. The inclusion of geometrical imperfections and material nonlinearity in the sections significantly results in further reducing the ultimate carrying capability of the columns.

Undoubtedly, the interaction among the different modes of behaviour is possible and coupled instabilities can be encountered in design [11,12]. Thus, it is essential for an engineer to have an in-depth understanding of the complex interactions involving local and global buckling, geometrical imperfections, material yielding and boundary conditions for the safe design of compressed thin-walled members.

**Figure 1:**
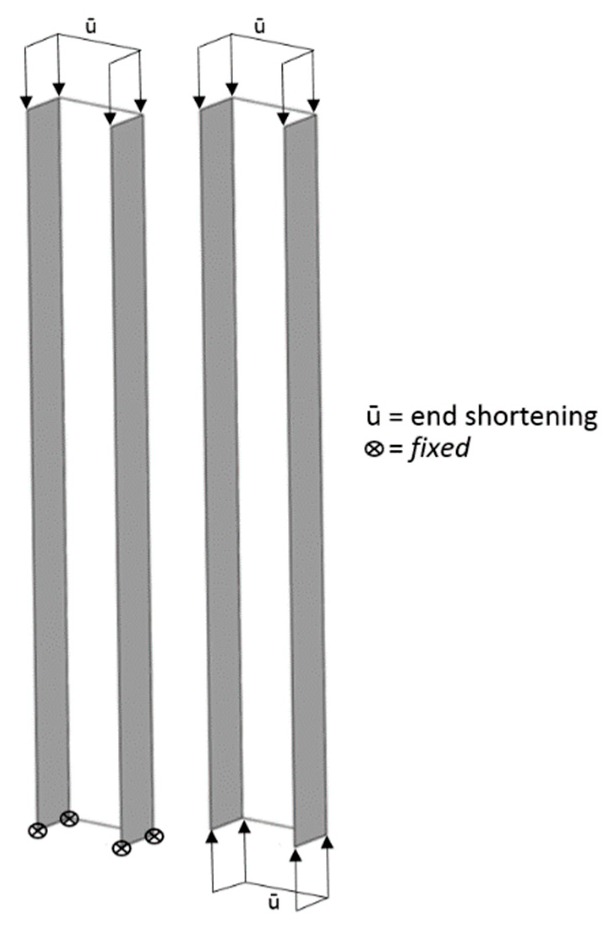
Forms of different loading conditions.

This paper details appropriate finite element modelling strategies and procedures using PATRAN/NASTRAN software to investigate the influence of loading conditions on the coupled local–overall/flexural interactive response of thin-walled singly-symmetric plain channel columns. The modelling procedures can describe the complete loading history of the compression members from the onset of local buckling through post-local buckling behaviour leading to local–overall/flexural interaction including material yielding and yield propagation to the ultimate conditions and then to elastoplastic unloading. The numerical simulations took into account the influence of material yielding on the compressive ultimate failures of the sections; however, the study was limited to the interaction of local buckling and overall flexural bending. Two forms of loading conditions were considered: the first was the one that has been used practically in tests whereby one end is loaded with a moving top platen while the other end is fixed at the lower platen, but, for the second loading condition, both ends were loaded with equally moving top and lower platens, as shown in Figure 1.

## 2. Finite Element Modelling

The nonlinear static solution sequence (SOL 600) was employed and used in the finite element method to simulate the compressional post-local buckling and overall bending interaction behaviour of the fixed-ended plain channel columns. Appropriate loading and boundary conditions as well as geometric nonlinearity and elastoplastic material nonlinearity using the simplified elastic–perfectly plastic stress–strain model were included in the simulation. The complete compressional loading history was determined from the beginning of the applied loading through the linear and nonlinear elastic and elastoplastic post-buckling period to ultimate plastic failure and then through the plastic unloading phase of behaviour. For the longer column members, some 30,000 elements and small load increments were used to accurately capture the complex post-local buckling and overall flexural bending interaction behaviour of the columns.

### 2.1. Geometry and Material Properties

**Figure 2:**
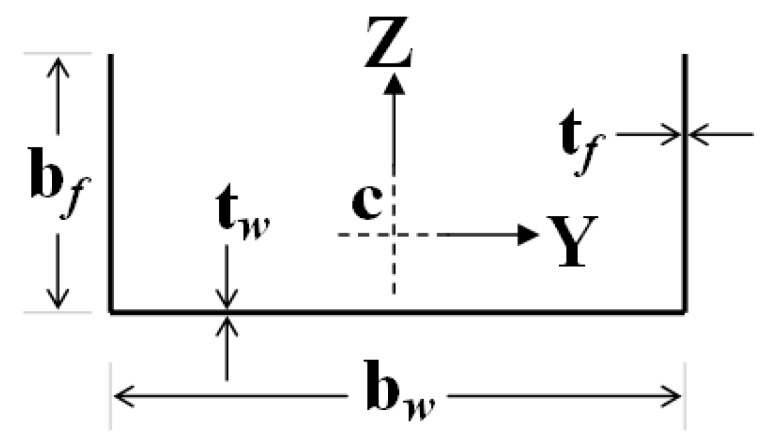
Geometrical details of the plain channel section.

The cross-section geometrical details for the channel-section columns are shown in Figure 2. The Z- and Y-axes are located at the section centroid with the *Z*-axis being in the plane of geometrical symmetry of the cross-section. The *Y*-axis is parallel with the web edge of the section whilst the *Z*-axis is parallel with the edge of the flanges. The *X*-axis pointing out of the paper is the longitudinal axis along the member length. The sections have a length *L*; the width of the web and flanges are denoted by *b_w_* and *b_f_*, respectively; and both the web and flanges have the thickness of *t_w_* and *t_f_*, as indicated. It is to be noted that the cross-section dimensions *b_w_* and *b_f_* are the mid-surface dimensions.

**Figure 3:**
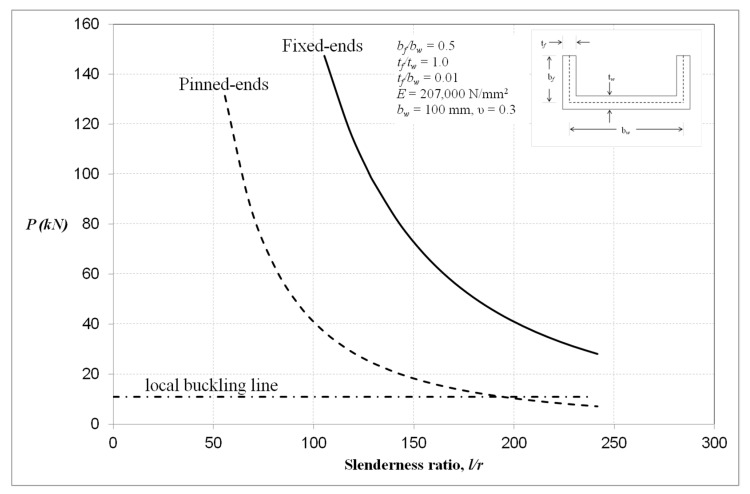
Euler curves for singly-symmetric plain channel column.

Linear buckling analysis was performed to determine the appropriate column length for examination. Figure 3 shows the local buckling line with pinned-ended and fixed-ended Euler curves of singly-symmetric plain channel section.

The findings are presented for typical singly-symmetric plain channel-section columns with *b_f_/b_w_* shape factor equal to 0.5. The width of the web, *b_w_*, is 100 mm and the thickness is 1.0 mm for both the flanges and the web. The column lengths of 700 and 1300 mm with corresponding slenderness ratios of 43 and 80, respectively, were chosen to represent short and long columns with only the local mode being present in the initial buckling. The material considered is steel with a Young’s modulus of 207,000 N/mm^2^ and a Poisson’s ratio of 0.3. From linear buckling analysis, the initial buckling mode shape showed that the column has local buckle crests at the column centre.

### 2.2. Loading and Boundary Conditions

The correct determination of the response of the columns to the applied loadings depends on the kinematic boundary conditions imposed on the finite element models. As mentioned above, the study was limited to the interaction of local buckling and overall flexural bending and thus the behaviour of overall torsional–flexural buckling was precluded from this study; consequently, symmetry conditions could be applied to the finite element models. For the case of being locally rotationally free at the plate end cross-section, only the out-of-plane movements were constrained, thus all flange nodes were constrained from moving in the Y-direction and the web nodes were prevented from movement in the Z-direction. In the case of locally rotationally constrained columns, for all nodes of the web and flanges at the end sections, apart from the out-of-plane displacement constraints, the out-of-plane rotations were constrained about the Y- and Z-axes, respectively. This paper is only concerned with the case of locally rotationally constrained columns.

**Figure 4:**
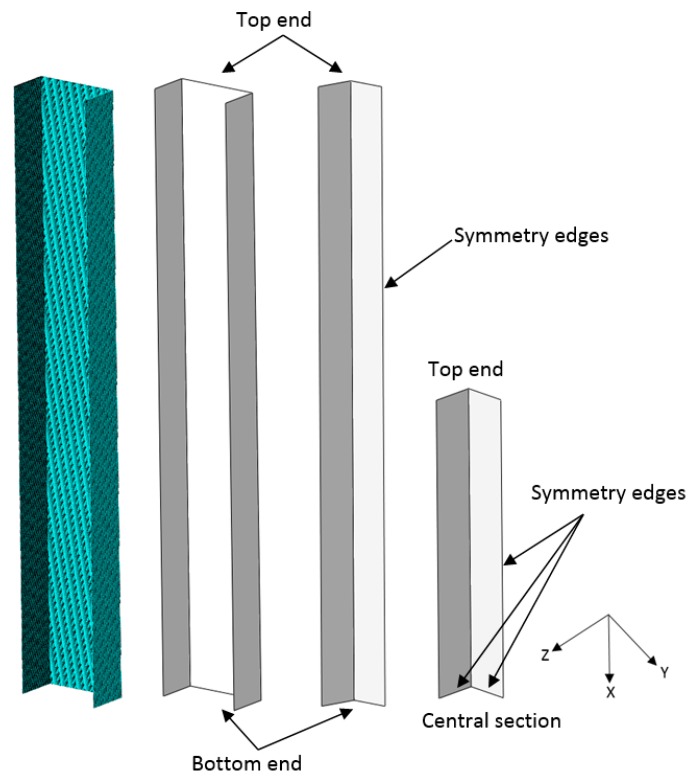
Plain channel sections (meshed full-model, full-model, half-model and quarter-model).

To simulate both types of loading conditions, CQUAD4 quadrilateral shell elements were used to discretise the sections and to formulate the finite element models, as shown in Figure 4. Three finite element models were used—full, half-section and quarter models—to simulate both types of loading conditions, as shown in Figure 4. It is worth pointing out that, although the full- and half-section models were the same, they were not practically the same as the quarter-model. 

In the case of the quarter-model, the application of loading was symmetrical where the model replicates a column with both ends being compressed and moving by the same amount. When considering the column quarter models, all nodes at the column central cross-section were constrained from movement in the X-direction with the flange nodes and the web nodes prevented from rotation about the *Y*-axis and about the *Z*-axis. When considering a column with a buckle crest existing at the column centre, this is reflected in the modelling process by zero rotation about the *Z*-axis at the flange nodes and zero rotation about the *Y*-axis at the web nodes. By using symmetry, only a portion of the actual structure needs to be modelled. The size of the finite element model—that is, the total number of nodes, elements and degrees-of-freedom—can be reduced, resulting in a significant reduction of the analysis run time and computer resources required by at least a factor of two. In addition, more accurate results can also be obtained by using a finer mesh in the reduced model than a comparable number of elements in a coarsely meshed full model. 

For the full- and half-section models, the loading was applied at one end while the other end was fixed and not moving. The nodes at one end of the cross-section were enforced to move by the same amount axially in the X-direction and those at the other end were not allowed to move axially. These conditions would have an effect at the centre cross-section of the column whereby the cross-sections of the full- and half-section models are moving towards the fixed end, but not with the quarter-model. Table 1 summarises the boundary conditions applied to the plain channel models.

### 2.3. Verification of Finite Element Model

**Figure 5:**
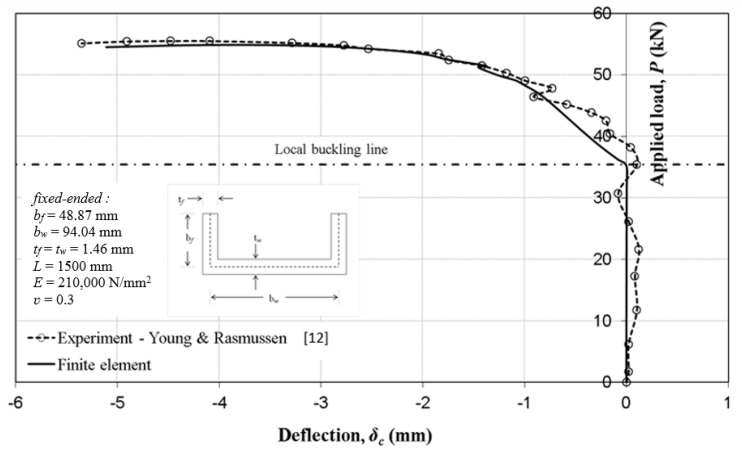
Column load–deflection comparisons with experimental tests.

**Figure 6:**
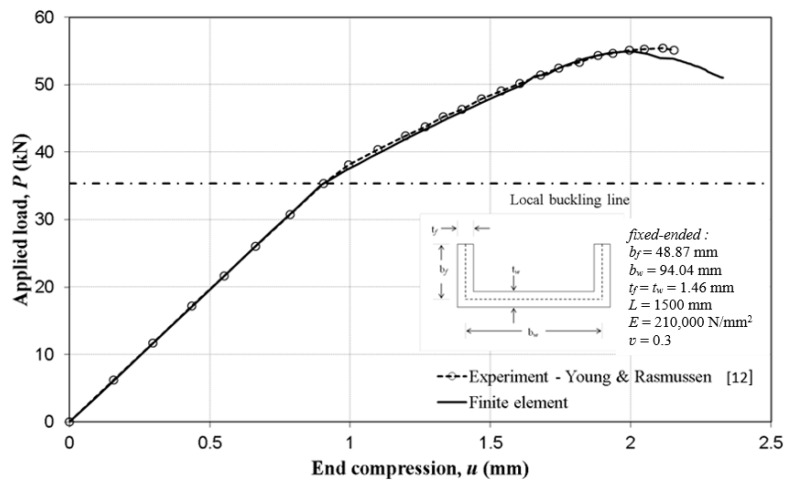
Load–end compression comparisons with experimental tests.

The behaviour of the fixed-ended plain channel column from the finite element simulation using the modelling strategies and solution procedures detailed in this paper was compared with the independent test work of Young and Rasmussen [12] and the comparison plots are shown in Figure 5 and Figure 6. The test column was 1500 mm long and the details of centreline dimensions of the section are as indicated in Figure 5. The actual stress–strain data reported by Young and Rasmussen [13] was used to represent the material model in the simulations, and the local geometric imperfection magnitude was in the order of *w_o_* = 0.01 mm. The column behaviour and the failure load from the simulation were seen to compare favourably with the test. The stable interaction was readily predicted and it is interesting to note that column deflection from the test began immediately after the local buckling load.

Figure 6 shows the applied load–end compression plot of the plain channel column. The column response to load from the simulation successfully followed that of the tested column. The compression history started from the beginning of the applied loading through linear and nonlinear elastic range and elastoplastic post-buckling period to ultimate plastic collapse and then through the plastic unloading phase of behaviour. The ultimate load of the simulation was in the order of 54.9 kN whilst that of the test was reported to be 55.5kN and the difference between the two is 1.08%.

## 3. Post-Buckling Responses

### 3.1. Plain Channel-Section Columns of Length 700 mm

**Figure 7:**
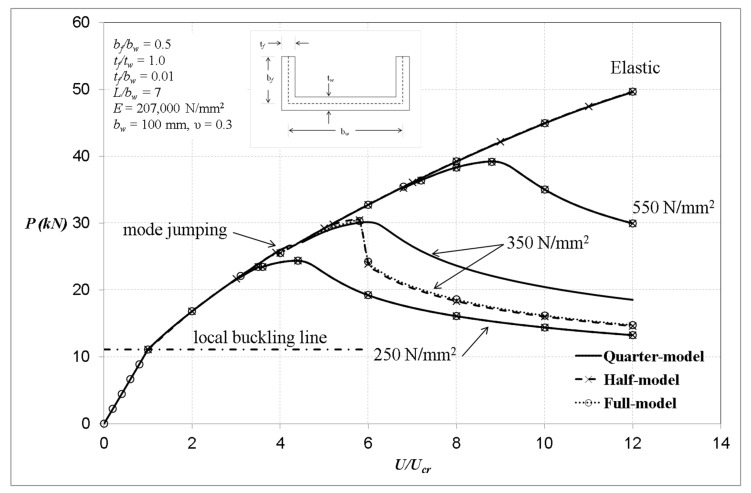
Columns with local buckle crest at column centre section (locally rotationally constrained).

The plain channel-section of length 700 mm with corresponding slenderness ratio of 43 can be categorised as short column. The finite element analyses with regards to those being local rotationally constrained at the plate ends are shown in Figure 7. The cross-sectional details are as indicated in the figure and the material considered is steel with a yield stress levels of 250, 350 and 550 N/mm^2^, Young’s modulus E = 207,000 N/mm^2^ and Poisson’s ratio *ν* = 0.3. The analyses were performed using perfect structural geometries with elastic and elastic–perfectly plastic material models.

The analyses were modelled using full-, half- and quarter-model to investigate the differences among these models. It can be noted that generally the quarter-model has good agreement with the full- and half-models except the one with a yield stress level of 350 N/mm^2^ of the locally rotationally constrained columns (Figure 7). It seems that, for the case of the singly-symmetric plain channel section, the quarter-model does not completely reproduce the same behaviour as the full- and half-models. The reason for this may be that the loading for the quarter-model was assumed to be applied by the same amount at both ends and the central column section does not move axially, whilst, for the case of the full- and half-models, the load was applied at one end and the other end was fixed and, in addition, the central column section does in fact move towards the fixed end. These factors, as shown below, caused the change in the number of local buckles along the length in a different way for all models and, thus, affected the post-buckling behaviour of the channel-section compression members.

In Figure 7, generally, it is clear that the fixed-ended column has a substantially stable post-local elastic interactive response. The ultimate loads were reduced considerably when taking into account the influence of material plasticity. The elastic ultimate load was in the order of 70 kN but was reduced to about 39 kN, 30 kN and 24.5 kN corresponding to the yield stress levels of σ_Y_ = 550, 350 and 250 N/mm^2^, respectively, in the elastoplastic solution. It is worth pointing out that, for the case where the channels are elastic, there is one point during the loading at which the numbers of local buckles change from five to seven for both cases of full- and quarter-models, and this is termed mode jumping, as indicated in Figure 7.

**Figure 8:**
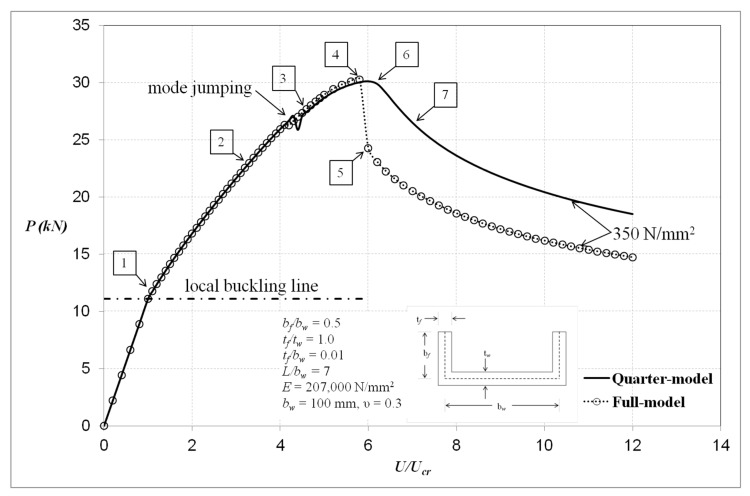
Load–end compression curves with yield stress level of 350 N/mm^2^.

**Figure 9:**
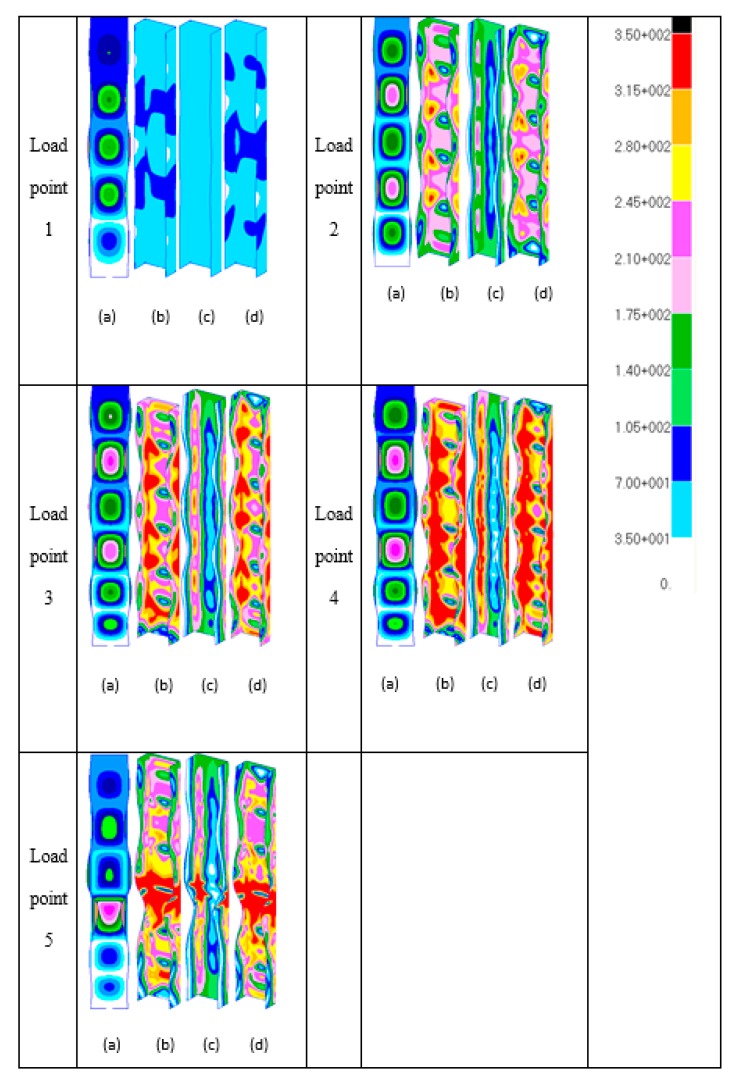
Deformation images (**a**) and growth of von Mises stresses with load ((**b**) outer surface 1; (**c**) middle surface; and (**d**) outer surface 2) of Figure 8 (full-model).

**Figure 10:**
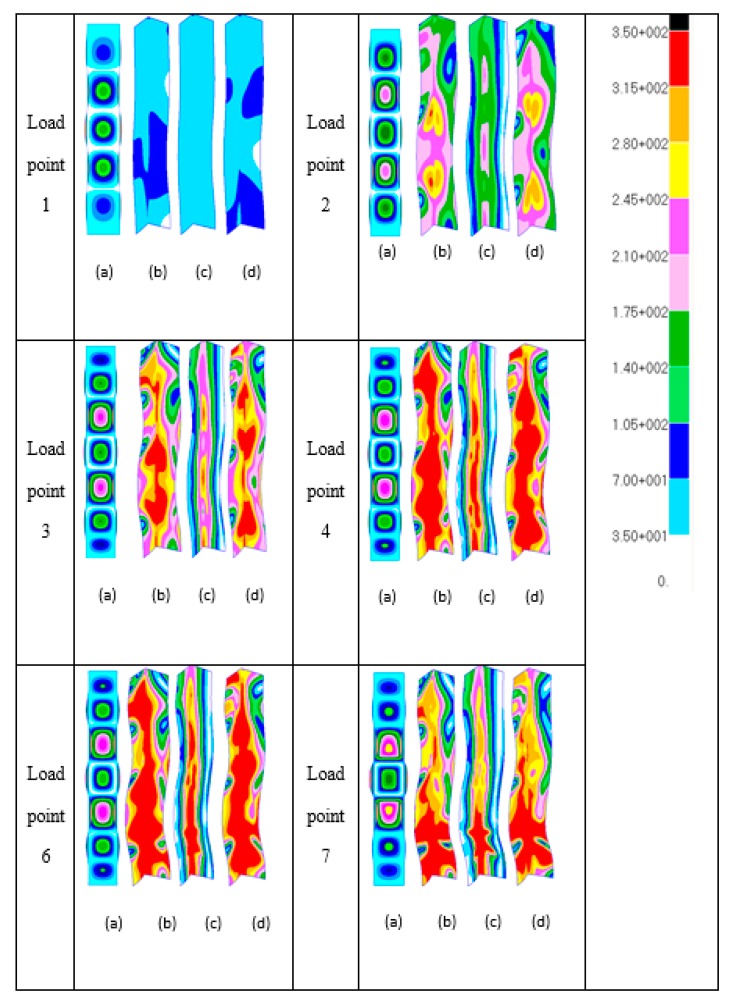
Deformation plots (**a**) and growth of von Mises stresses with load ((**b**) outer surface 1; (**c**) middle surface; and (**d**) outer surface 2) of Figure 8 (quarter-model).

**Figure 11:**
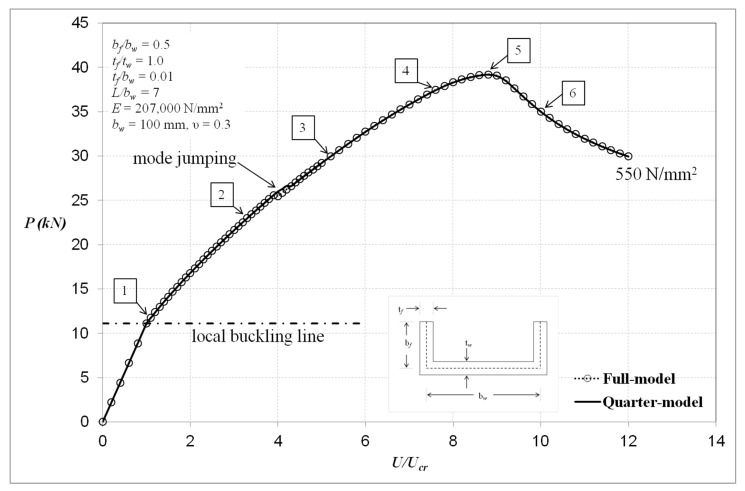
Load–end compression curves with yield stress level of 550 N/mm^2^.

**Figure 12:**
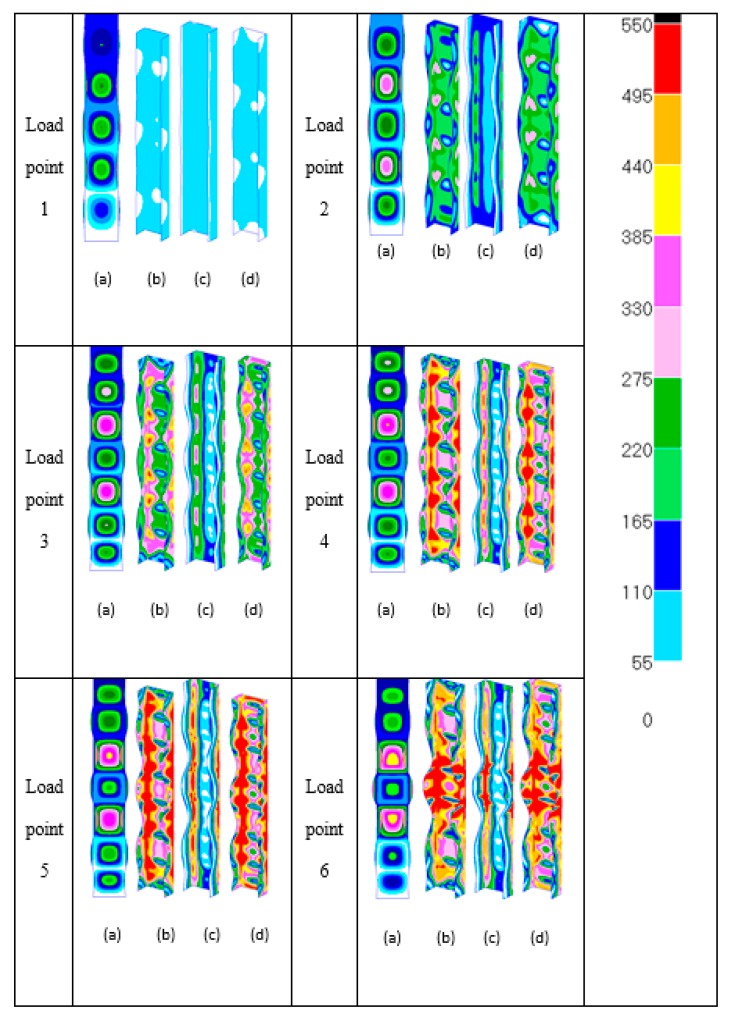
Deformation plots (**a**) and growth of von Mises stresses with load ((**b**) outer surface 1; (**c**) middle surface; and (**d**) outer surface 2) of Figure 11 (full-model).

It can be seen in Figure 7 that the behaviours of the full- and half-models are similar, and that the quarter-model almost replicates the same responses as the full- and half-models when the material is elastic. However, once plasticity is taken into consideration, near the ultimate condition and related to the yield stress level of 350 N/mm^2^, the behaviour of the columns using the full-model tends to change unexpectedly at the point of the ultimate load, while responses of those using the quarter-model are gradually unloaded after reaching the failure load. Nevertheless, all models virtually predict the same final collapse load. Interestingly, for the yield stress levels of 250 and 550 N/mm^2^, the quarter-model produces similar behaviour to the full- and half-models, and with further examination the full-model shows symmetric deformation to have occurred with the plane of symmetry located at the central column section, as would be expected when using the quarter-model. More in-depth investigation on the deformation shapes and the development of von Mises stresses with particular interest in the columns behaviour with yield stress levels of 350 and 550 N/mm^2^ are shown in Figure 8, Figure 9, Figure 10, Figure 11 and Figure 12.

Figure 8 shows the equilibrium behaviour curves of the full- and quarter-models. It can be noted that the slope of both curves diminishes as the load increases due to material yielding and local buckled form change during compression. Both models show comparable responses up to the point of the collapse load. Upon reaching the ultimate load, the equilibrium response of the compression member of the full-model suddenly drops, while that of the quarter-model steadily unloads. Hence, there is a need to investigate further what triggered the different responses. The deformation images and the development of von Mises stresses of the full- and quarter-models in relation to the load locations of Figure 8 are shown in Figure 9 and Figure 10, respectively.

Figure 9 depicts images of the deformed shapes and growth of von Mises stresses using the full-model. At load point location 1, the von Mises stresses are everywhere elastic, which is less than the material yield stress level of *σ_Y_* = 350 N/mm^2^, as indicated in Figure 9. At load level 2, the maximum stress is seen to be 339 N/mm^2^. For the section considered with *b_f_/b_w_* = 0.5, it is found that first yielding takes place between load levels 2 and 3, as indicated in Figure 9. It can be seen from the deformed shape at load level 2 that the member has five local buckles. At load level 3, from the deformation image, the number of local buckles jumps from five to six unequal local buckles where the central local buckle has been shifted slightly upwards. At this load level, von Mises yielding at *σ_Y_* = 350 N/mm^2^ is seen to occur in the web and flange elements of the section on outer surfaces 1 and 2 at the section nodal planes and in the vicinity of the section junction. It is clear from this that complete yielding through wall thickness has almost been reached at the locations between nodal planes and in the vicinity of the section junction, as shown in the middle surface image of load level 3. At load level 4, it is obvious that yield propagation has taken place through the section wall thickness and along parts of the section junctions mostly in the lower part of the column on all surfaces. It is seen that the section junction in this region in particular is now fully yielded. Load level 4 is essentially representing the ultimate conditions of the section prior to the unloading phase of its compressional behaviour. At load level 5, it is clear that there are significant plastic yield hinges located at the lower nodal plane of the central local buckle. From the observations made with respect to yield propagation with load, it is perhaps most relevant to point out that, at the ultimate load condition pertaining to load levels 3 and 4, it is clear that failure is closely associated with complete through-the-thickness yielding at the section junctions on part of the section length. This is first evident at load level 4, as indicated in Figure 9, due to asymmetric conditions of the local buckles along the length and it is of some significance that failure ensues shortly thereafter at load level 5, as shown in Figure 9.

Figure 10 depicts images of the deformed shapes and progress of von Mises stresses using the quarter-model. Bear in mind that the quarter-model represents a complete symmetry of the finite element model where the boundary conditions along with the applied loads are all symmetric. The deformation images in that case were constructed by reflecting the images of the quarter-model at the planes of symmetry and thus the images in Figure 10 represent the full length of the channel from end to end. The von Mises stress images correspond to half the column length. These stress images were taken from the nonlinear finite element analyses without any modification and in isometric view to show the significant development of von Mises stresses in the vicinity of the section junctions.

From the deformation images, at load points 1 and 2, there are five local buckles. As the applied load increases at one point between load points 2 and 3, the number of local buckles changes from five to seven and this is depicted in the deformed shape of load point 3. It can be noted that the mode jumping happens in a symmetric manner and the crest of middle local buckle does not shift from the central column line. For the case of the full-model, however, it is believed that the change in number of local buckles was affected by simultaneous yielding and mode jumping. Moreover, the channel still has the reserve strength to pass the critical mode jumping point before failure. This observable fact was investigated further by examining the equilibrium curves with yield stress level of 550 N/mm^2^, whereby the quarter-model produces similar behaviour to that of the full-model.

It is seen that, at load point location 1, the von Mises stresses are everywhere elastic, as indicated in Figure 10. At load level 2, the maximum stress increases to a level close to von Mises yielding at *σ_Y_* = 350 N/mm^2^, and it is believed that first yielding takes place between load levels 2 and 3, as indicated in Figure 10. At load level 3, von Mises yielding is seen to occur in the web and flange elements of the section on outer surface 1 and 2 at the section nodal planes and in the vicinity of the section junction. At load point 4, it is clear from this that complete yielding through the wall thickness has been reached in the vicinity of the section junction close to the column central section. Load point 6 essentially represents the ultimate plastic failure of the column and from the observations made it is perhaps worth noting that, at the ultimate load condition pertaining to load levels 6 and 7, it is clear that failure is closely associated with complete through-the-thickness yielding at the section junctions close to the column central section.

Figure 11 shows the load–end compression equilibrium behaviour of the plain channel with respect to yield stress level 550 N/mm^2^. Based on Figure 11, for sections manufactured from high yield materials, it is possible to design the columns with a fairly wide range of the ratio of yield stress to local buckling stress *σ_Y_*/*σ_cr_* of the sections. For the higher values of this ratio, it is shown that the post-buckling response involves an initial range of elastic behaviour whereby the nonlinear loss in compressional stiffness is associated with large rotations and local form change during loading.

Based on the results in Figure 11, more in-depth investigation is required since the full-model generates similar responses to that of the quarter-model. The deformation shapes and development of von Mises stresses relating to the column behaviour at each number shown on the end compression plot of Figure 11 are depicted in Figure 12.

At load point locations 1 and 2, the von Mises stresses are everywhere elastic as predicted since the compression material is in the initial stages of post-buckling. At load level 3, the maximum stress is seen to be 522 N/mm^2^. Again, this is seen to occur on outer surface 1 and on the nodal planes in the vicinity of the section junction. It is noticed that, from the deformation images of load levels 2 and 3, the number of local buckles jumps from five to seven and this mode jumping location is indicated in Figure 11. The additional local buckle is seen to be equally placed below and in the upper central region of the column. It is found that first yielding happens between load levels 3 and 4, where it has passed the critical point of mode jumping. At load level 4, von Mises yielding at *σ_Y_* = 550 N/mm^2^ is seen to expand mostly in the web and flange elements of the section on outer surfaces 1 and 2 of the same spot as at load level 3. It is clear from this that yielding has also taken place through wall thickness between the nodal planes of each local buckle half-wavelength and in the vicinity of the section junction. At load level 5, it is evident that yield propagation has taken place through the section wall thickness predominantly along the section junction close to its mid-height. Load level 5 essentially represents the ultimate conditions of the section prior to the unloading phase of its compressional behaviour. At load level 6, it is clear that there is significant yield propagation on the middle surface all along the section junction between the nodal planes of central local buckle. From the observations made with respect to yield propagation with load, it is perhaps most relevant to point out that failure is closely associated with complete through-the-thickness yielding at the section junctions close to the section central column length.

It is worth pointing out that for, the case of a yield stress level of 250 N/mm^2^, the first von Mises yielding takes place at an applied load level of about 21.5 kN, and it is seen that the compressional behaviour of the column deviates from the elastic equilibrium curve just before the local mode change of the elastic locally buckled column. It should be noted also that there is a local mode change along the column length at an applied load level of about 24.4 kN, whereby the number of local buckles changes from five to seven for both cases of full- and quarter-models.

Generally, the compressional stiffness of thin-walled short columns is noted to be significantly changed as a result of the out-of-plane buckles of the section walls. The presence of surface yielding, which is due to the high through-the-thickness bending stresses, has caused more loss in compressional stiffness, and the growth of yielding through the section wall thickness leads finally to ultimate plastic collapse and then to a post-collapse unloading phase of behaviour. It is of note that at ultimate conditions von Mises yielding is seen creeping into the middle surface from the outer surfaces mostly in the vicinity of the section junctions along the column length.

For the particular case of short columns locally rotationally constrained at their plate ends, at one point during loading, the number of local buckles changes and it perhaps depends mostly on the loading conditions and the start of von Mises yielding. The post-collapse behaviour is noted to be significantly altered as a result of local mode change along the column length. For additional numbers of local buckles equally placed below and in the upper regions of the column mid-height, the post-collapse phase of behaviour is observed to be gradually unloading and it is seen that the failure mechanism occurs at the centre of the column. For the unsymmetrical arrangement of the local buckles along the column length, upon following the ultimate conditions, the column crumples at a location slightly off the column mid-height and the load rapidly drops to a level way below the failure load.

### 3.2. Plain Channel-Section Columns of Length 1300 mm

**Figure 13:**
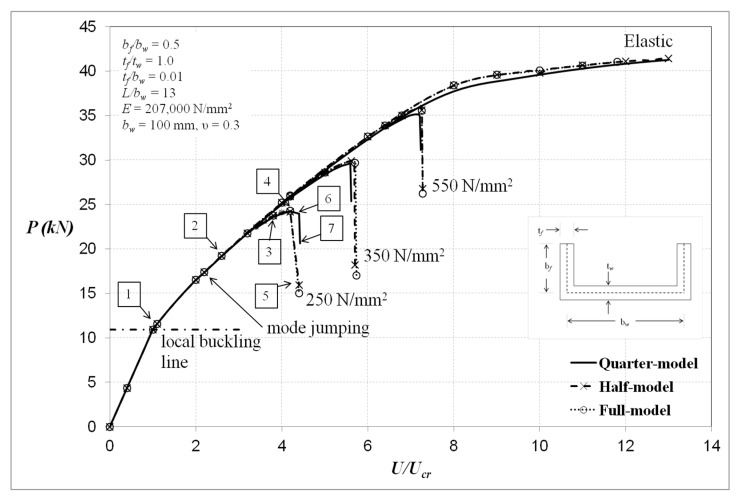
Load–end compression curves (locally rotationally constrained).

Figure 13 shows the axial load, end-compression characteristics for plain channel columns of length 1300 mm that are locally rotationally constrained at the plate ends. In the elastic range, the fixed-ended column shows a substantially stable post-local interactive response from the onset of local buckling and that the ultimate carrying capability of the column is of the order of 41.6 kN. The influence of material plasticity has caused the elastic ultimate load to be reduced considerably. The elastic ultimate load is seen to be reduced to about 36 kN, 30 kN and 24 kN for the yield stress levels of *σ_Y_* = 550, 350 and 250 N/mm^2^, respectively, in the elastoplastic solutions. The reduction based on the lower yield stress levels of 250 N/mm^2^ is noted to reduce the elastic ultimate load of the column in the order of 42.3%.

The nonlinear finite element analyses of Figure 13 employed three finite element models, the full-, half- and quarter-models. The three models basically estimate the same failure loads; however, the post-collapse behaviours are quite different. As the loading increases, the compression stiffness of the quarter-model is found to be lower than those of the other two models. Perhaps the two most influential factors are the slenderness ratio of the columns and the distribution of local buckles along the column length. It is of note that for the case of locally rotationally constrained columns, the phenomenon of mode jumping is present during compression and this alters the numbers and the distribution of the local buckles along the length. Figure 13 shows that the elastic curve of the quarter-model deviates from that of the full-model at *P* = 27 kN. Hence, it is necessary to examine the cause of these differences by looking at the deformed states and the growth of the von Mises stresses for the full- and quarter-models.

**Figure 14:**
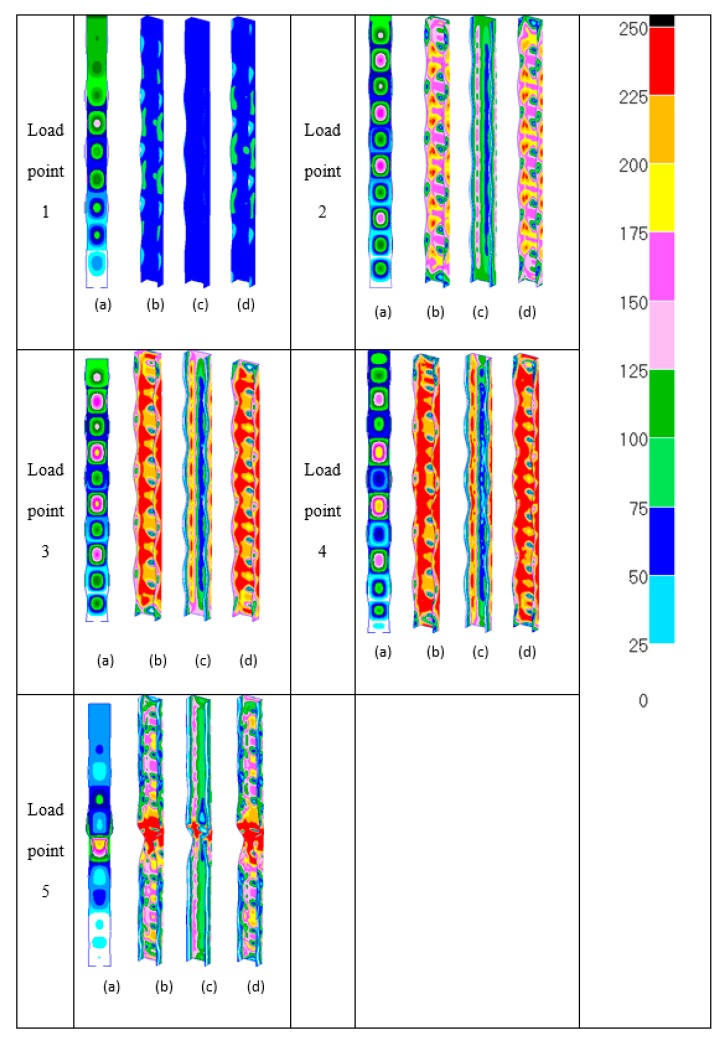
Deformation plots (**a**) and the growth of the von Mises stresses with load ((**b**) outer surface 1; (**c**) middle surface; and (**d**) outer surface 2) of Figure 13 (full-model).

The development of the deformed shapes and the von Mises stresses corresponding to the end compression plot of Figure 13 using the full finite element model is shown in Figure 14. The numbers shown in Figure 14 indicate the load locations in relation to a yield stress level of 250 N/mm^2^. In Figure 14, the spreading of plastic yielding with load and the most probable mechanism of failure at plastic collapse can be ascertained. In this case, the out-of-plane rotations at the ends of the constituent plate elements are constrained and, as a result, the local buckle amplitudes diminish towards the ends of the column. This can be seen in the locally buckled mode shape at load location 1 whereby the column is seen to be perfectly straight and the local buckles along the length are associated with amplitude modulation with the maximum amplitude located at the column centre.

At load point locations 1 and 2, the von Mises stresses are everywhere less than the material yield stress level of *σ_Y_* = 250 N/mm^2^. At load level 2, the maximum stress is seen to be 240 N/mm^2^. For the section considered, the first yielding is found to occur between load levels 2 and 3, as indicated in Figure 14. It should be noted also that there is a local mode change along the column length at applied load level 2 whereby the number of local buckles changes from 9 to 10 and this mode jumping location is indicated in Figure 13. The additional local buckle is seen to be positioned below the central region of the column and also the central local buckle has been pushed upwards to make spaces for the additional local buckles. As a result, the region below the column mid-height has greater numbers of local buckles than the upper region. At load level 3, von Mises yielding occurs in the web and flange elements of the section on outer surfaces 1 and 2 and in the vicinity of the section junction along the length.

It is clear from this that complete yielding through the wall thickness has been reached at locations between the nodal planes and in the vicinity of the section junction at load level 3. At load level 4, it is obvious that full yield propagation has taken place along the section junction on all surfaces and that the section junction in particular is now fully yielded. Essentially, load level 4 represents the ultimate conditions of the section prior to the unloading phase of its compressional behaviour. At load level 5, it is clear that plastic mechanisms take place in the region below the central local buckle due to the fact that heavier local buckles are located in the lower region.

**Figure 15:**
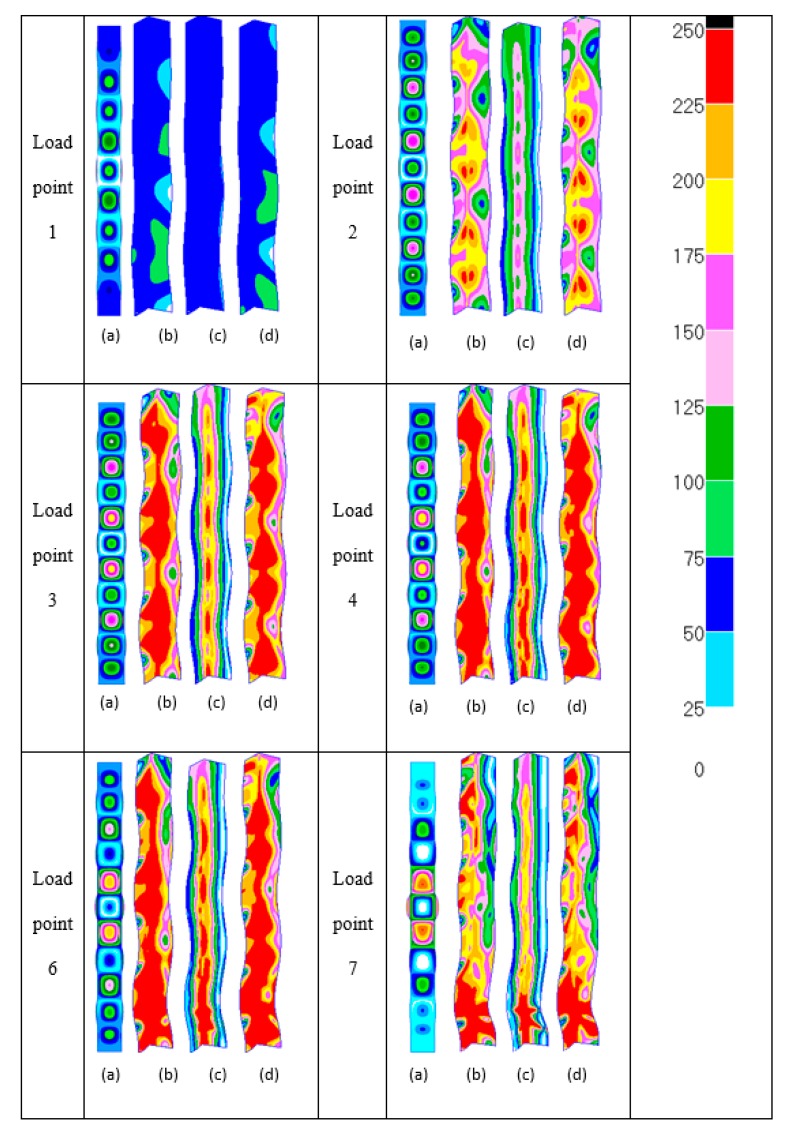
Deformation plots (**a**) and the growth of the von Mises stresses with load ((**b**) outer surface 1; (**c**) middle surface; and (**d**) outer surface 2) of Figure 13 (quarter-model).

The growth of the deformed shapes and the von Mises stresses in relation to the end compression plot of Figure 13 using the quarter finite element model is shown in Figure 15. The von Mises stresses are everywhere elastic at load point locations 1 and 2, as indicated in Figure 15. At load level 2, the maximum stress is seen to be 244 N/mm^2^ on outer surfaces 1 and 2, which is close to the von Mises yielding at *σ_Y_* = 250 N/mm^2^. The first yielding is expected to occur between load levels 2 and 3, as indicated in Figure 15. It is noticed that, from the deformation images of load levels 1 and 2, the number of local buckles jumps from 9 to 11, and this mode jumping location is indicated in Figure 13. The additional local buckle is seen to be equally placed below and in the upper central region of the column. At load level 3, von Mises yielding occurs in the web and flange elements of the section on outer surfaces 1 and 2 and in the vicinity of the section junction along the length. It is clear from this that complete yielding through the wall thickness has been reached at different spots between the nodal planes and in the vicinity of the section junction. At load levels 4 and 5, the ultimate condition has been attained as a result of the full yield spreading on all the surfaces along the section junction near the column’s central region. At load level 5, it is clear that plastic mechanisms take place between the nodal planes of the central local buckle and in the proximity of the section junctions due to the fact that heavier local buckles are equally placed in the lower and upper region of the column.

**Figure 16:**
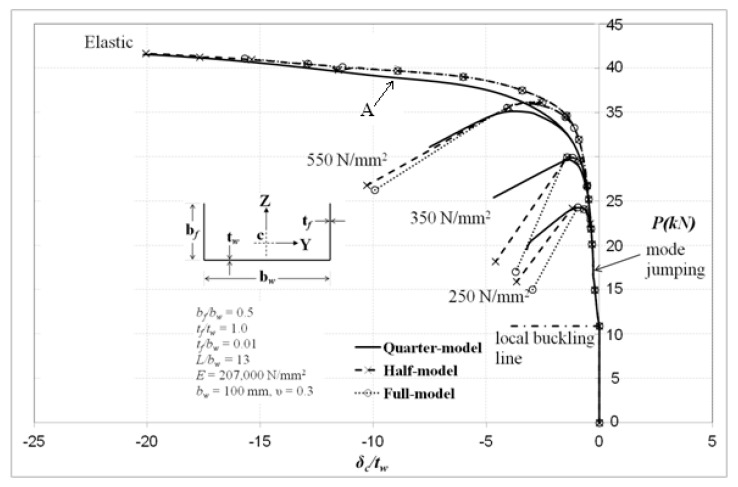
Load–deflection curves (locally rotationally constrained).

**Figure 17:**
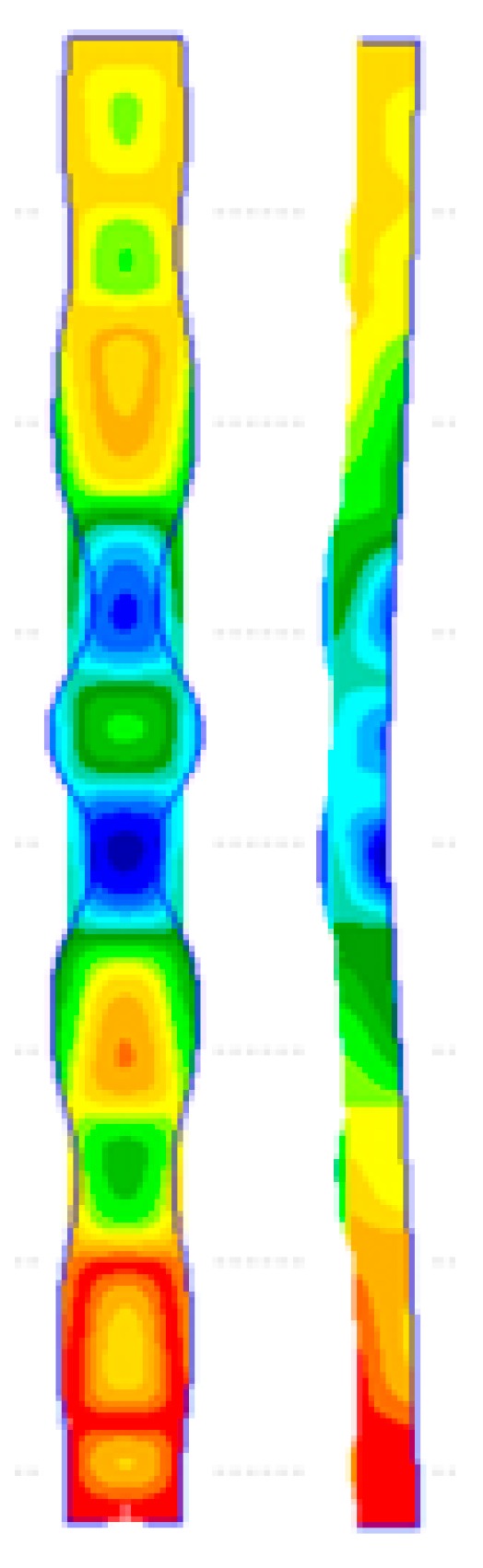
Deformation plots at location A.

Figure 16 shows the axial load and the column central deflection characteristics for the plain channel columns of Figure 13, whereby the global flexural mode is clearly evident. In the elastic range of behaviour, the local–overall deflected state of the column at location *A* is seen to have heavier local buckles in the central region in which the flange’s free edges are under the greatest compression, whereas the web element of the section is more heavily compressed in the outer region and the amplitude of the local buckles is seen to diminish towards the column ends, as depicted in Figure 17.

It should also be noted that there is a local mode change along the column length at an applied load level of about 17 kN, whereby the number of local buckles changes from 9 to 11 for the quarter-model, whereas for the case of the full-model the local mode changes from 9 to 10 local buckles. This mode jumping location is indicated in Figure 16. These changes could be the reason the compression stiffness of the full-model is different from that of the quarter-model. As the number of local buckles increases, as in the case of the quarter-model, this constitutes more loss in the effective area and thus the column becomes ineffective in resisting further loading.

## 4. Concluding Remarks

The modelling strategies and procedures employed in the finite element simulations for the correct determination of the failure mechanics of plain channel section columns are described in this paper. For this particular case of fixed-ended, plain channel columns with locally-rotationally constrained plate ends, it was shown that the behaviour of the singly-symmetric plain channel columns changed due to the mode jumping in the buckling mode as a result of the different types of loading conditions. The results presented in this paper provide deep insight and understanding of the complete loading history of the columns and show the development of yielding throughout loading. The post-buckled compressional stiffness was shown to be altered due to the change in the number of buckles along the columns and this also finally resulted in reducing the ultimate failure of the columns. This can be seen for the longer columns with higher yield stresses.

## Figures and Tables

**Table 1:** Summary of boundary conditions.

DOF	Top End		Bottom End		Symmetry Edges		
	Web Nodes	Flange Nodes	Web Nodes	Flange Nodes	Column Central Section (Buckle Crest)	Column Central Section (Buckle Node)	Column Half Section
Ux	ū	ū	R	R	R	R	F
Uy	F	R	F	R	F	F	R
Uz	R	F	R	F	F	F	F
URx	F	F	F	F	F	F	R
URy	R	F	R	F	R (web nodes only)	R	F
URz	F	R	F	R	R (flange nodes only)	R	F

Notes: F and R denote “*free*” and “*restrained*”, respectively. ū is the applied end shortening.
